# Associations Between Daily Mood States and Brain Gray Matter Volume, Resting-State Functional Connectivity and Task-Based Activity in Healthy Adults

**DOI:** 10.3389/fnhum.2018.00168

**Published:** 2018-05-01

**Authors:** Elmira Ismaylova, Jessica Di Sante, Jean-Philippe Gouin, Florence B. Pomares, Frank Vitaro, Richard E. Tremblay, Linda Booij

**Affiliations:** ^1^Research Center, Sainte-Justine hospital, Montreal, QC, Canada; ^2^Department of Psychiatry, University of Montreal, Montreal, QC, Canada; ^3^Department of Psychology, Concordia University, Montreal, QC, Canada; ^4^School of Psychoeducation, University of Montreal, Montreal, QC, Canada; ^5^Department of Psychology and Pediatrics, University of Montreal, Montreal, QC, Canada; ^6^School of Public Health, Physiotherapy and Sports Science, University College Dublin, Dublin, Ireland

**Keywords:** daily mood, fMRI, left hippocampus, default-mode network, emotion

## Abstract

Numerous studies have shown differences in the functioning in the areas of the frontal-limbic circuitry between depressed patients and controls. However, current knowledge on frontal-limbic neural substrates of individual differences in mood states in everyday life in healthy individuals is scarce. The present study investigates anatomical, resting-state, and functional neural correlates of daily mood states in healthy individuals. We expected to observe associations between mood and the frontal-limbic circuitry and the default-mode network (DMN). A total of 42 healthy adults (19 men, 23 women; 34 ± 1.2 years) regularly followed for behavior and psychosocial functioning since age of 6, underwent a functional magnetic resonance imaging scan, and completed a daily diary of mood states and related cognitions for 5 consecutive days. Results showed that individuals with smaller left hippocampal gray matter volumes experienced more negative mood and rumination in their daily life. Greater resting-state functional connectivity (rsFC) within the DMN, namely between posterior cingulate cortex (PCC) and medial prefrontal cortex regions as well as between PCC and precuneus, was associated with both greater negative and positive mood states in daily life. These rsFC results could be indicative of the role of the DMN regional functioning in emotional arousal, irrespective of valence. Lastly, greater daily positive mood was associated with greater activation in response to negative emotional stimuli in the precentral gyri, previously linked to emotional interference on cognitive control. Altogether, present findings might reflect neural mechanisms underlying daily affect and cognition among healthy individuals.

## Introduction

The function and structure of frontal-limbic brain regions play a major role in the regulation of mood. Most of the evidence stems from anatomical and functional magnetic resonance imaging (fMRI) studies conducted in individuals with major depressive disorder (MDD). Compared to healthy controls, individuals with MDD displayed smaller gray matter (GM) volume in such regions as dorsal lateral prefrontal cortex (LPFC) (e.g., Shad et al., 2012; Grieve et al., 2013) and hippocampus (e.g., Zou et al., 2010; Stratmann et al., 2014). Individuals with MDD also display greater neural responses to negative emotional stimuli in limbic regions including amygdala and hippocampus (e.g., Victor et al., 2010; Hall et al., 2014), as well as lower resting-state functional connectivity (rsFC) between amygdala and such (pre)frontal regions as dorsal LPFC, ventral medial prefrontal cortex (MPFC), and anterior cingulate cortex (ACC) (e.g., Pannekoek et al., 2014; Connolly et al., 2017). Additionally, several studies indicated that MDD is characterized by resting-state functional hypoconnectivity between dorsal LPFC and parietal regions, which are involved in attending to the environmental cues, as well as hyperconnectivity among MPFC, ACC, and hippocampus, implicated in self-referential processes (e.g., Kaiser et al., 2015; Northoff, 2016). In MDD, this connectivity “imbalance” would contribute to shifting focus on self-oriented thoughts, potentially resulting in rumination (e.g., Kaiser et al., 2015; Northoff, 2016).

While MDD-related maladaptive affect and cognition has been associated with altered frontal-limbic brain processes, transient changes in mood can also transiently alter neural functioning in these networks. This has been demonstrated by experimental studies in healthy individuals, combining fMRI with experimental mood-induction techniques such as emotional videos/images/music and autobiographical recall of emotional events (e.g., Harrison et al., 2008; Subramaniam et al., 2016). In healthy adults, experimental induction of negative mood has been associated with heightened brain activity in amygdala and hippocampus and various prefrontal regions including the orbitofrontal cortex (OFC), MPFC, ventral LPFC, and ACC, as well as with lowered rsFC between posterior cingulate cortex (PCC) and MPFC and greater ACC-insula rsFC (e.g., Pelletier et al., 2003; Habel et al., 2005; Harrison et al., 2008). Induction of positive mood in healthy adults has been associated with greater neural activity in MPFC, dorsal LPFC, and PCC (e.g., Habel et al., 2005; Subramaniam et al., 2016).

Several fMRI studies have investigated the neural correlates of mood reactivity in daily life using ecological assessment methods. Most of these studies focused on patients with psychotic disorders or at-risk samples. For instance, one brain morphometry study showed that (relative to controls) greater emotional reactivity to daily stress in patients with schizophrenia and at-risk first-degree relatives was associated with smaller hippocampal GM volumes (Collip et al., 2013). Using task-based fMRI, Tully et al. (2014) indicated that within individuals with schizophrenia (relative to controls), higher dorsal LPFC activity during cognitive control of negative emotional information was associated with positive mood following highly distressing interpersonal conflict. In a study of couples free of mental health problems, Hooker et al. (2010) showed that lower ventral LPFC response to negative emotional stimuli was associated with greater daily negative mood and rumination following stressful interpersonal conflict with their partner.

Additionally, individual differences in rsFC have been linked to the well-being/positive lifestyle, such as life satisfaction, self-realization, or pleasure attainment (Smith et al., 2015; Luo et al., 2017). Noteworthy, PCC-based rsFC with PFC, precuneus, parahippocampal, and superior temporal gyri – all part of the so-called default-mode network (DMN) (Greicius et al., 2003; Fransson, 2005, 2006; Buckner et al., 2008) – has been linked to experimentally induced sadness (Zamoscik et al., 2014; Renner et al., 2017). We are not aware of any study examining the association between these brain processes and the daily-life mood in healthy individuals. The link between PCC-based rsFC and daily mood is of particular interest since it might be informative of how mood states in the everyday life echo in the functioning of the brain not strained by any specific task.

The aim of the present study was to examine, in a sample of healthy adults, anatomical, resting-state, and functional neural correlates of daily mood states, namely negative mood, rumination, and positive mood. We expected these associations to occur in brain regions that are part of the frontal-limbic neural circuitry and the DMN, namely prefrontal cortex, anterior and posterior cingulate cortices, precuneus, insula, hippocampus, and amygdala.

## Materials and Methods

### Participants

Participants were members of two longitudinal cohorts of individuals, followed since their kindergarten year (Van Bokhoven et al., 2006; Rouquette et al., 2014). Careful screening for eligibility in the brain-imaging component was based on the absence of any prior or current Axis I disorder, neurological disorder, medical illness, and medication intake as well as on the absence of irremovable foreign metals in the body (e.g., braces, piercings). Presence/absence of past and current Axis I disorders was assessed by conducting the Structured Clinical Interview for DSM-IV (SCID-IV) (First et al., 2002), whereas the other (neuroimaging-related) exclusion criteria were verified by means of a Montreal Neurological Institute (MNI) in-house questionnaire. After thorough screening for eligibility and availability, 47 individuals underwent an fMRI session at the MNI. Participants also completed the Beck Depression Inventory (BDI) (Beck et al., 1988) as well as the Neuroticism scale (12 items) of the Eysenck Personality Questionnaire (EPQ) (Birley et al., 2006), neither of which have been found to be associated with any brain processes in the current study. After the fMRI session, participants were asked to fill out a daily online questionnaire for five consecutive days.

Five participants did not fill out the daily online questionnaire despite multiple reminders. Therefore, the final sample of the present study was composed of 42 healthy adults (19 men, 23 women; age range 32–36 years). Written informed consent was obtained from all participants in accordance with the Declaration of Helsinki (World Medical Association, 1991). The study protocol was approved by the ethics review boards of Sainte-Justine Hospital and of MNI, Montreal, Canada.

### Image Acquisition

All 42 participants were scanned on a 3T Siemens TIM Trio Scanner^1^ using a 32-channel head-coil. Following a brief localizer, the scan sequence included, respectively, a 9-min-long anatomical scan (MPRAGE sequence; 176 slices in sagittal plane; TR = 2300 ms, TE = 2.98 ms, FA = 9°, FOV = 256 mm, matrix size = 256 × 256, voxel size = 1 mm × 1 mm × 1mm), a 7-min-long resting state scan (gradient EPI sequence; 180 whole-brain volumes; TR = 2300 ms, TE = 30 ms, FA = 30°, FOV = 224 mm, matrix = 64 × 64, 43 slices in axial plane, voxel size = 3.5 mm × 3.5 mm × 3.5 mm) during which participants were asked to stay awake while keeping their eyes closed (Beliveau et al., 2015), and lastly a 15-min-long event-related functional scan during which an emotion-processing task was performed (gradient EPI sequence; 400 whole-brain volumes; TR = 2300 ms, TE = 30 ms, FA = 30°, FOV = 224 mm, matrix = 64 × 64, 43 slices in axial plane, voxel size = 3.5 mm × 3.5 mm × 3.5 mm). During the emotion-processing task, adapted from Canli et al. (2005), 120 Ekman facial emotional expressions (happy, sad, angry, fearful, and neutral) (Ekman and Frisen, 1976; Keltner and Ekman, 2003) were presented to participants randomly for 2 s, followed by a fixation cross (1 s) and a question asking participants to choose whether the face belonged to a man or a woman. Activation following exposure to happy (versus neutral) and sad (versus neutral) was studied in the context of the present study.

### Daily Diary of Mood States

Following the brain scan, participants were instructed to fill out online a daily diary of mood states, at the end of the day for five consecutive days. This online diary allowed participants to provide precise information on the mood they have experienced in their natural context (e.g., family life, marital life, social life), within the 24-h period for 5 consecutive days. All the responses were in the form of multiple choices, ranging from “never” to “repeatedly.”

Positive and Negative Affect subscales were formed based on the Positive And Negative Affect Schedule (PANAS) (Watson et al., 1988), assessing the extent to which participants had experienced, respectively, positive emotional states characterizing sharp and alert mind (happy, enthusiastic, energetic, determined) and negative emotional states with negative connotations (irritable, sad, nervous, embarrassed) over a period of 24 h, in a form of a multiple choice ranging from “never” (score “0”) to “repeatedly” (score “4”). Therefore, the total score for each scale ranged from 0 to 16.

Repetitive negative thoughts (rumination) were assessed with the items of the Ruminative Responses Scale of the Response Style Questionnaire (RSQ) (Nolen-Hoeksema et al., 1999). Relative to the reflective pondering subtype of rumination (involving active attempts to gain insight into problems), brooding subtype of rumination (implying passive comparison of one’s current situation with an unachieved standard), and worry have been consistently associated with negative mood in healthy and clinical populations (e.g., Burwell and Shirk, 2007; Raes and Hermans, 2008; Verhaeghen et al., 2014). Therefore, we constructed a subscore focusing solely on the brooding and worry components, as potential contributors to the maintenance and intensification of mood states. Eight RSQ-derived items describing brooding rumination and worry were used for the construction of the current scale (i.e., “Negative thoughts came to mind throughout the day,” “I analyzed recent events to try to understand why I was sad or upset,” “I wondered why I always react this way,” “I wondered why I cannot seem to cope better with events,” “I imagined how I would have liked things to happen,” “I worried about saying or doing something wrong,” “I worried about what others would think of me,” and “ I worried about being criticized for something I said or did”). For each statement, the participants were asked to indicate how many times they felt this way in a form of a multiple choice, ranging from “never” (score “0”) to “repeatedly” (score “3”). Therefore, the total score for this scale ranged from 0 to 24.

Total scores for each subscale were averaged for the period of five consecutive days, indicating individuals’ average experience of daily mood, and used in the analyses (Bolger et al., 2003). Cronbach’s alpha for the subscales of the daily diary averages across 5 days were 0.99 for negative mood, 0.91 for rumination, and 0.99 for positive mood, indicating excellent internal stability.

### Statistical Analyses

Statistical Parametric Mapping [(SPM12) v6470, Wellcome Department of Cognitive Neurology, London, United Kingdom] implemented in MATLAB R2010a (Mathworks, Sherborne, MA, United States) was used for anatomical and functional analyses.

Voxel-based morphometry (VBM) analyses were computed using the CAT12 toolbox^2^. T1-weighted images were normalized to MNI space and segmented into GM, white matter (WM), and cerebrospinal fluid (CSF) based on intensity distribution of the image and using tissue probability maps. Then, normalized GM segments were modulated with the resulting Jacobian determinant maps and smoothed with an 8-mm full width at half maximum (FWHM) Gaussian kernel. VBM, not biased to one particular structure, allows a balanced comprehensive assessment of anatomical differences throughout the brain (Ashburner and Friston, 2001). Whole-brain analysis was conducted, followed by a region of interest (ROI) analysis using a mask encompassing frontal-limbic regions (PFC, ACC, insula, hippocampus, and amygdala) obtained from Anatomical Automatic Labeling (AAL) atlas within the Wake Forest University Pick Atlas utility (version 3.0.5) (Maldjian et al., 2003, 2004). For the anatomical analyses, the brain regions were examined at a voxel-wise threshold of *p* < 0.001, with cluster-wise family-wise error (FWE)-correction for multiple comparisons (Shaffer, 1995) threshold set at *p* < 0.05, and corrected for smoothness non-uniformity (Hayasaka et al., 2004). All peak coordinates are reported in MNI format.

Using CONN functional connectivity toolbox (version 16.b) pipeline (Whitfield-Gabrieli and Nieto-Castanon, 2012) and based on SPM12 preprocessing, resting-state functional images were spatially realigned to correct for interscan movement and normalized to the MNI space, while structural images were segmented and separately normalized to the MNI space with non-linear transformation. Next, ART-based scrubbing was done, which consisted in a detection of outlier functional scans (defined as points exceeding the default threshold set with a conservative 95% percentile, including a global brain activation signal of *z* > 3 and linear motion parameters > 0.5 mm). Any outlier functional scans were, then, entered as a covariate during the denoising step to control for potential confounding effect. Finally, all the images were smoothed using an 8-mm FWHM Gaussian kernel. A denoising procedure including the component-based correction method (Behzadi et al., 2007) followed by the first-level analysis was applied in order to remove motion artifacts and other artifactual effects from the fMRI signal. Next, considering that PCC-based – one of the DMN’s nodes (Greicius et al., 2003) – connectivity has been previously associated with mood states (e.g., Zamoscik et al., 2014; Renner et al., 2017), second-level seed-based analysis was conducted using PCC as a seed, which was defined using AAL atlas (MNI coordinates: -6, -52, 40) (Fox et al., 2005). For the connectivity analyses, the brain regions were examined at a voxel-wise threshold of *p* < 0.001, with cluster-wise FWE-correction for multiple comparisons (Shaffer, 1995) threshold set at *p* < 0.05. All peak coordinates are reported in MNI format. When significant results were observed, the individual connectivity values for the regions showing up in the seed-to-voxel analysis were extracted using the CONN v.16.b software. These connectivity values were, then, transferred in an Excel document in order to generate a scatter-plot for visualization purposes.

Preprocessing of the functional data acquired during the emotion-processing task (slice timing, realignment, co-registration, stereotaxic spatial normalization, and smoothing with 8-mm FWHM Gaussian kernel) was followed by intra-individual first-level analyses, performed in order to calculate (emotion minus neutral) contrasts for each emotion at each voxel. Next, whole-brain analysis was performed, followed by ROI analysis using a mask encompassing all frontal-limbic regions (see earlier) (Maldjian et al., 2003, 2004). For the functional analyses, the brain regions were examined at a voxel-wise threshold of *p* < 0.001, with cluster-wise FWE-correction for multiple comparisons (Shaffer, 1995) threshold set at *p* < 0.05. All peak coordinates are reported in MNI format.

We performed multiple regression VBM and fMRI analyses, with separately negative mood, rumination, and positive mood as second-level variables in the imaging analyses. The same variables of interest were separately implemented to test for voxel-wise correlations between them and PCC-based resting-state connectivity. A total of nine analyses were performed in the present study, for which we applied the cluster-wise FWE correction for multiple comparisons.

## Results

### Descriptive Analyses

**Table 1** shows the characteristics of the included sample. BDI and EPQ Neuroticism scale scores were generally considered low compared to normative scores (Beck et al., 1988; Birley et al., 2006). As expected, daily negative mood was found to be positively correlated to daily rumination (*r* = 0.73, *p* < 0.001). Daily positive mood was not found to be significantly correlated to either daily negative mood (*r* = -0.24, *p* = 0.12) or daily rumination (*r* = -0.29, *p* = 0.06). Furthermore, BDI scores (as assessed at the time of scanning) were found to be positively correlated to daily negative mood (*r* = 0.45, *p* = 0.003) and daily rumination (*r* = 0.32, *p* = 0.04). In the same vein, EPQ Neuroticism scale scores were found to be positively correlated to daily negative mood (*r* = 0.51, *p* = 0.001) and daily rumination (*r* = 0.40, *p* = 0.009). No significant association was found between daily positive mood and BDI score and EPQ Neuroticism scale score (*p* > 0.11). There were no significant associations between daily mood and sex [*F*(1,40) = 0.10–1.60; *p* > 0.213].

**Table 1:** Characteristics of the sample.

Characteristics of the sample (*N* = 42)	Statistics			
Variable	Mean	*SD*	Median	Skewness
Age (years)	33.95	1.15	34.00	0.189
Sex (Male/Female)	19/23	–	–	–
EPQ Neuroticism scale score (/12)	2.45	2.40	2.00	0.680
BDI total score (/63)	3.26	3.44	2.00	0.717
Daily negative mood score (/16)	2.35	1.46	2.20	0.907
Daily rumination score (/24)	3.10	2.74	2.00	1.058
Daily positive mood score (/16)	9.00	2.60	8.80	0.126

*SD = Standard deviation; EPQ = Eysenck Personality Questionnaire; BDI = Beck Depression Inventory.*

### Imaging Analyses

#### Brain Morphometry

**FIGURE 1:**
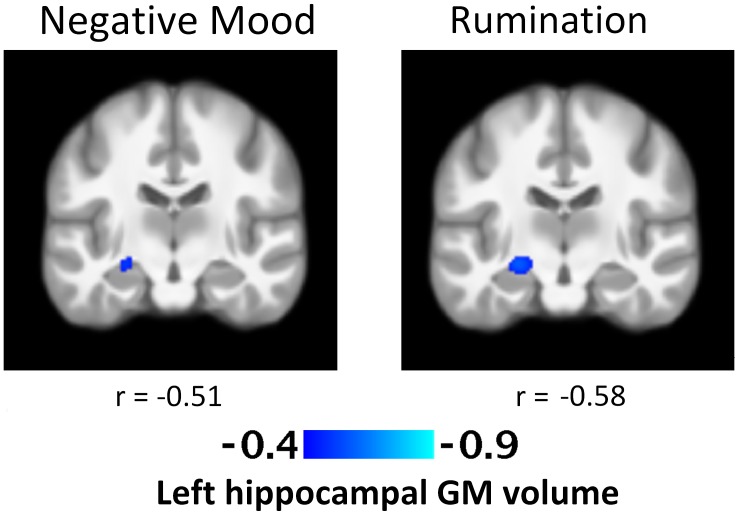
T-Statistic maps of the negative association between regional left hippocampal gray matter volume and daily negative mood and rumination. Both images were taken at peak Montreal Neurological Institute coordinates: –24, –18, –9 and presented at the whole-brain level of voxel *p* < 0.001. There was no significant association after FWE correction.

No significant associations were found at the voxel *p* < 0.001 level following the cluster-wise FWE-correction. The exploratory results of positive and negative VBM GM correlations significant at the uncorrected voxel *p* < 0.001 level without the cluster-wise FWE-correction are indicated in **Table 2**. Greater negative mood in daily life was associated with smaller left hippocampal GM volume [*k* = 110; *t* = 4.02; cluster *p* = 0.16; voxel *p* < 0.001; (peak MNI coordinates: -24, -18, -9)] (**Figure 1**). Greater levels of rumination were also associated with smaller left hippocampal GM volume [*k* = 215; *t* = 4.54; cluster *p* = 0.06; voxel *p* < 0.001; (peak MNI coordinates: -24, -18, -9)] (**Figure 1**). No other significant associations were found.

**Table 2:** Exploratory gray matter findings of whole-brain voxel-based morphometry analyses and their associations with daily mood states.

						MNI peak coordinates		
Variable	Region	*k*	Voxel *t*	Cluster *p*-value^∗^	Voxel *p*-value^∗^	*x*	*y*	*z*
Negative mood	left hippocampus	110	-4.02	0.157	< 0.001	-24	-18	-9
Rumination	left hippocampus	215	-4.54	0.061	< 0.001	-24	-18	-9

*MNI = Montreal Neurological Institute. ^∗^Uncorrected for multiple comparisons.*

#### Resting-State Functional Connectivity

**FIGURE 2:**
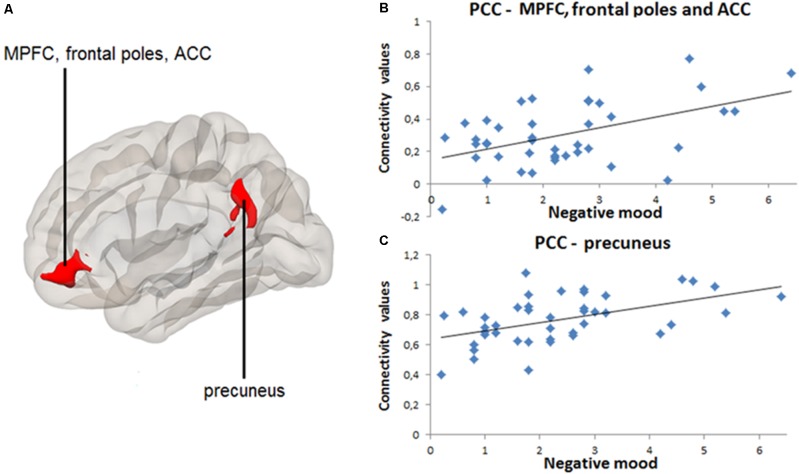
Positive correlation between daily negative mood and resting-state connectivity between posterior cingulate cortex (PCC) and medial prefrontal cortex (MPFC), frontal poles, anterior cingulate cortex (ACC), as well as between PCC and precuneus. PCC-based seed-to-voxel connectivity 3D map presented using CONN toolbox. **(A)** Left red cluster depicts MPFC, frontal poles, and ACC positively coupled with PCC. Right red cluster depicts precuneus positively coupled with PCC. **(B)** Scatter-plot for visual inspection illustrates the result from the extracted mean connectivity values of the left cluster within the default-mode network (DMN). Data was taken at peak Montreal Neurological Institute (MNI) coordinates: –4, 40, –6; *k* = 609; cluster *p_FWE_*< 0.001. **(C)** Scatter-plot for visual inspection illustrates the result from the extracted mean connectivity values of the right cluster within the DMN. Data was taken at MNI coordinates: –6, –54, 34; *k* = 939; cluster *p_FWE_*< 0.001.

**FIGURE 3:**
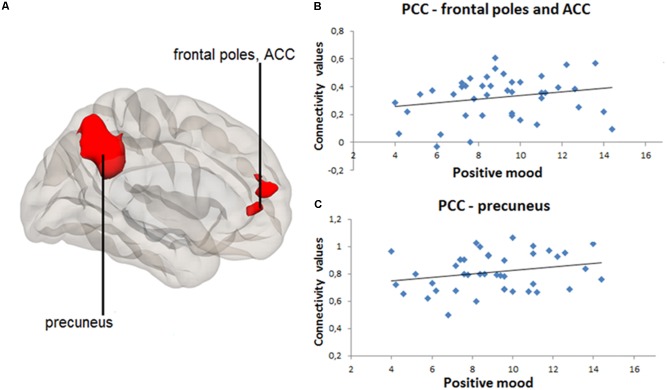
Positive correlation between daily positive mood and resting-state connectivity between posterior cingulate cortex (PCC) and frontal poles and anterior cingulate cortex (ACC), as well as between PCC and precuneus. PCC-based seed-to-voxel connectivity 3D map presented using CONN toolbox. **(A)** Right red cluster depicts frontal poles and ACC positively coupled with PCC. Left red cluster depicts precuneus positively coupled with PCC. **(B)** Scatter-plot for visual inspection illustrates the result from the extracted mean connectivity values of the right cluster within the default-mode network (DMN). Data was taken at peak Montreal Neurological Institute (MNI) coordinates: 2, 56, 12; *k* = 437; cluster *p_FWE_*< 0.001. **(C)** Scatter-plot for visual inspection illustrates the result from the extracted mean connectivity values of the left cluster within the DMN. Data was taken at MNI coordinates: –10, –50, 38; *k* = 1980; cluster *p_FWE_*< 0.001.

Significant results of positive and negative seed-to-voxel rsFC correlations are indicated in **Table 3**. Greater daily negative mood was associated with greater rsFC between PCC and MPFC, frontal poles and ACC [*k* = 609, *t* = 4.64, *p_FWE_* < 0.001, voxel *p* < 0.001, (peak MNI coordinates: -4, 40, -6)], as well as between PCC and precuneus [*k* = 939, *t* = 5.02, *p_FWE_* < 0.001, voxel *p* < 0.001, (peak MNI coordinates: -6, -54, 34)] (**Figure 2A**). **Figures 2B,C** contain scatter-plots depicting how the connectivity values of the PCC-MPFC, frontal poles, ACC cluster, and PCC-precuneus cluster vary with daily negative mood. Daily positive mood was also associated with greater seed-to-voxel rsFC between PCC and frontal poles and ACC [*k* = 437, *t* = 4.57, *p_FWE_* < 0.001, voxel *p* < 0.001, (peak MNI coordinates: 2, 56, 12)], as well as between PCC and precuneus [*k* = 1980, *t* = 6.77, *p_FWE_* < 0.001, voxel *p* < 0.001, (peak MNI coordinates: -10, -50, 38)] (**Figure 3A**). **Figures 3B,C** contain scatter-plots depicting how the connectivity values of the PCC-frontal poles, ACC cluster and PCC-precuneus cluster vary with daily positive mood. No other significant associations between daily mood and PCC-based rsFC were found.

**Table 3:** Results of whole-brain seed-to--voxel resting-state functional connectivity analyses and their associations with daily mood states.

							MNI peak coordinates		
Variable	Seed region	Target cluster	*k*	Voxel t	Cluster *p_FWE_*-value	Voxel *p*-value	*x*	*y*	*z*
Negative mood	PCC	MPFC, frontal poles and ACC	609	4.64	< 0.001	< 0.001	-4	40	-6
	PCC	Precuneus	939	5.02	< 0.001	< 0.001	-6	-54	34
Positive mood	PCC	Frontal poles and ACC	437	4.57	< 0.001	< 0.001	2	56	12
	PCC	Precuneus	1980	6.77	< 0.001	< 0.001	-10	-50	38

*MNI = Montreal Neurological Institute; FWE = Family-wise error; PCC = posterior cingulate cortex; MPFC = medial prefrontal cortex; and ACC = anterior cingulate cortex.*

#### Task Imaging Results

Greater daily positive mood was associated with greater bilateral precentral responses to sad stimuli, respectively, for right precentral gyrus: [*k* = 82; *t* = 4.13; cluster *p_FWE_* = 0.016; voxel *p* < 0.001; (peak MNI coordinates: 8, -21, 63)] and left precentral gyrus [*k* = 82; *t* = 3.87; cluster *p_FWE_* = 0.02; voxel *p* < 0.001; (peak MNI coordinates: -6, -21, 60)]. No other significant associations were found.

## Discussion

In the present study, we examined neural correlates of daily mood states in healthy adults. Daily negative and positive mood were each associated with greater rsFC between PCC and such regions as ACC and precuneus. Furthermore, positive mood was associated with greater precentral responses to negative (i.e., sad) emotional stimuli. Lastly, we also found that daily negative mood and rumination were each associated with smaller left hippocampal GM volume (albeit only at the voxel-wise threshold of *p* < 0.001). Overall, these results seem to indicate not only mood-state-specific neural correlates but also that natural variation in daily mood is reflected in the frontal-limbic functioning and anatomy, relevant for understanding subtle changes in daily emotional life among healthy individuals.

In line with the previous research showing that individuals scoring higher in such positive personal indicators as positive affect and lifestyle satisfaction exhibited stronger rsFC patterns within the DMN including MPFC and parietal cortex (Smith et al., 2015), positive associations were observed between daily positive mood and DMN resting-state connectivity between PCC and MPFC regions. Additionally, the finding that both positive and negative daily mood states were linked to the rsFC within the same DMN regions may suggest that heightened PCC-MPFC rsFC could reflect an arousal (as opposed to valence) dimension of mood. In other words, individuals with increased PCC-MPFC functional connectivity at rest might experience more emotions on a daily basis, irrespective of valence. Further studies are necessary to replicate these findings as well as to examine potential physiological confounds, including heart rate.

Smaller left hippocampal GM structure has been repeatedly associated with various psychiatric symptoms, including trauma, MDD, and schizophrenia (e.g., Seidman et al., 2002; Vythilingam et al., 2002; Steffens et al., 2011). Smaller right hippocampal GM volume has been repeatedly associated with childhood maltreatment and combat-related woes, particularly in individuals who later developed post-traumatic stress disorder (e.g., Bremner et al., 1995; PaviČ et al., 2007). We may advance that in the present context, subtle variations in hippocampal GM volume – although on an uncorrected level – may be associated with individual differences in daily negative mood and ruminative response style in healthy adults. However, further studies are necessary to confirm these exploratory uncorrected results.

Lastly, a positive association was found between daily positive mood and brain activation in response to negative (i.e., sad) emotional stimuli in the precentral gyri. In addition to its role in motor behavior, heightened activity in precentral gyri – distinct structure in the posterior portion of the frontal lobe – has also been implicated in the retrieving and processing of (positive or negative) emotionally valenced stimuli (Maratos et al., 2001; Kolesar et al., 2017). Worth noting, precentral regional activity has also been positively associated with emotional interference in cognitive and behavioral control, condition in which one’s attention and goal-directed behavior is challenged by emotionally salient stimuli (see meta-analysis by Song et al., 2017). Overall, current task-based activity in the precentral gyri (that are bound inferiorly by the cingulate cortices) as well as PCC-ACC resting-state connectivity may echo brain mechanisms involved in the assessment and cognitive regulation of emotions in everyday life. In light of these findings, current low between-subject scores and variation in daily negative mood and rumination, relative to daily positive mood, may reflect degrees of adaptive emotional reactivity and regulation in a healthy sample.

Strength of the study was that the current participant sample was part of a well-documented longitudinal sample followed for more than 3 decades, and participants for the present study were carefully selected on the basis of absence of psychopathology. This permitted to avoid any (residual) clinical symptoms or treatment as a potential confound. On the other hand, the careful screening for mental health problems may have led to low inter- and intra-individual differences in mood and low levels of negative affect and rumination. Additionally, the number of statistical tests may have increased the probability of finding false positives. Yet on the other hand, the relatively moderate sample size combined with a stringent testing could result in increasing probability of type II errors. Despite a restricted range in daily mood and a moderate sample size, significant associations were detected suggesting that individual differences in daily affect and cognition among healthy individuals are reflected in individual differences in frontal-limbic and DMN brain processes. Future research should include larger samples with a large range of daily mood levels and could include possible moderating factors such as genotype and sex.

In summary, this study showed in a sample of healthy adults that individual differences in left hippocampus GM volumes were associated with individual differences in negative affect in daily life. The observed association between both daily positive and negative mood and PCC-MPFC and precuneus rsFC might be indicative of the involvement of this DMN regional functioning in the emotional arousal in daily life, irrespective of valence. Future research should include samples with a larger inter- and intra-individual range of daily mood states.

## Author Contributions

EI collected the data, performed the analyses, and wrote the manuscript. JDS assisted in the data acquisition and analyses. FP and LB supervised the brain imaging analysis. J-PG designed the daily mood diary and provided assistance in the data analyses. JDS, FP, FV, J-PG, and RT revised the manuscript critically. RT and FV initiated the longitudinal cohort in whom the study was conducted. LB designed the study and supervised and revised the manuscript critically.

## Conflict of Interest Statement

The authors declare that the research was conducted in the absence of any commercial or financial relationships that could be construed as a potential conflict of interest.

## Footnotes

1 www.medical.siemens.com

2 http://www.neuro.uni-jena.de
